# Comparing the excepted values of atom-bond connectivity and geometric–arithmetic indices in random spiro chains

**DOI:** 10.1186/s13660-018-1628-8

**Published:** 2018-02-17

**Authors:** Shouliu Wei, Xiaoling Ke, Guoliang Hao

**Affiliations:** 1 Department of Mathematics, Minjiang University, Fuzhou, P.R. China; 2 College of Science, East China University of Technology, Nanchang, P.R. China

**Keywords:** 05C05, 05C12, *ABC* index, *GA* index, Spiro chain, Average value, Comparison

## Abstract

The atom-bond connectivity (*ABC*) index and geometric–arithmetic (*GA*) index are two well-studied topological indices, which are useful tools in QSPR and QSAR investigations. In this paper, we first obtain explicit formulae for the expected values of *ABC* and *GA* indices in random spiro chains, which are graphs of a class of unbranched polycyclic aromatic hydrocarbons. Based on these formulae, we then present the average values of *ABC* and *GA* indices with respect to the set of all spiro chains with *n* hexagons and make a comparison between the expected values of *ABC* and *GA* indices in random spiro chains.

## Introduction

A connected graph with maximum vertex degree at most 4 is said to be a *molecular graph*. Its graphical representation may resemble a structural formula of some (usually organic) molecule. That was a primary reason for employing graph theory in chemistry. Nowadays this area of mathematical chemistry is called *chemical graph theory* [1]. Molecular descriptors play a significant role and have found wide applications in chemical graph theory especially in investigations of the quantitative structure-property relations (QSPR) and quantitative structure-activity relations (QSAR). Among them, topological indices have a prominent place [2]. There exists a legion of topological indices that have some applications in chemistry [2, 3]. One of the best known and widely used topological indices is the connectivity index (Randić index) introduced in 1975 by Randić [4], who has shown that this index can reflect molecular branching. Some results on molecular branching can be found in [5–9] and the references therein. However, many physico-chemical properties depend on factors rather different from branching.

All graphs considered in this paper are simple, undirected, and connected. The notation not defined in this paper can be found in the book [10]. Let *G* be a graph with vertex set $V(G)=\{v_{1}, v_{2},\dotsc, v_{n}\}$ and edge set $E(G)$. Denote by $d_{i}$ the degree of the vertex $v_{i}$ in *G*. If an edge connects a vertex of degree *i* and a vertex of degree *j* in *G*, then we call it an $(i,j)$*-edge*. Let $m_{ij}(G)$ denote the number of $(i,j)$-edges in *G*.

In 1998, Estrada et al. [11] proposed a topological index of a graph *G*, known as the *atom-bond connectivity index*, which is abbreviated as $\mathit{ABC}(G)$ and defined as

1 $$ \mathit{ABC}(G)=\sum_{v_{i}v_{j}\in E(G)}\sqrt{ \frac{d_{i}+d_{j}-2}{d_{i}d_{j}}}, $$

where the summation goes over all edges of *G*. The *ABC* index has been proven to be a valuable predictive index in the study of the heat of formation in alkanes and has been applied up to now to study the stability of alkanes and the strain energy of cycloalkanes [11, 12]. For some recent contributions on the *ABC* index, we refer to [13–17].

As an analogue to the *ABC* index, a new topological index of a graph *G*, named the *geometric–arithmetic index* and abbreviated $\mathit{GA}(G)$, was considered by Vukićević and Furtula [18] in 2009. The *GA* index is defined as follows:

2 $$ \mathit{GA}(G)=\sum_{v_{i}v_{j}\in E(G)}\frac{2\sqrt{d_{i}d_{j}}}{d_{i}+d_{j}}, $$

where the summation goes over all edges of *G*. It is noted in [18] that the *GA* index is well correlated with a variety of physico-chemical properties and the predictive power of *GA* index is somewhat better than the Randić index. Up to now, many mathematical properties of *GA* index were investigated in [15, 19–23] and the references therein.

**Figure 1:**
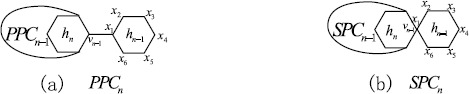
A polyphenyl chain $\mathit{PPC}_{n}$ and the corresponding spiro chain $\mathit{SPC}_{n}$ with *n* hexagons

**Figure 2:**
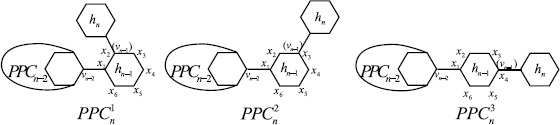
Three types of local arrangements in a polyphenyl chain

Polyphenyls and their derivatives, which can be used in organic synthesis, drug synthesis, heat exchanger, and so on, attracted the attention of chemists for many years [24–26]. A *polyphenyl chain* of length *n* is obtained from a sequence of hexagons $h_{1}, h_{2}, \dots, h_{n}$ by adding a cut edge to each pair of consecutive hexagons, which is denoted by $\mathit{PPC}_{n}$. The hexagon $h_{i}$ is called the *ith hexagon* of $\mathit{PPC}_{n}$ for $1\le i\le n$. Figure 1(a) shows a general polyphenyl chain, where $v_{n-1}$ is a vertex of $h_{n-1}$ in $\mathit{PPC}_{n-1}$. Note that, there are three ways to add a cut edge between two consecutive hexagons. So $\mathit{PPC}_{n}$ is not unique when $n\ge3$. Let $h_{n-1}=x_{1}x_{2}x_{3}x_{4}x_{5}x_{6}$ in $\mathit{PPC}_{n-1}$ for $n\ge3$. There is a cut edge connecting $x_{1}$ and $v_{n-2}$, which is a vertex in $h_{n-2}$. By symmetry there are three ways to add a cut edge between the $(n-1)$th hexagon $h_{n-1}$ of $\mathit{PPC}_{n-1}$ to the extra hexagon $h_{n}$. Precisely, let $\mathit{PPC}_{n}^{1}$, $\mathit{PPC}_{n}^{2}$, and $\mathit{PPC}_{n}^{3}$ be the graphs obtained by adding a cut edge connecting a vertex of the extra hexagon $h_{n}$ with vertex $x_{i+1}$ of $h_{n-1}$ (see Figure 2), where $i=1,2,3$. Many results on matching and independent set, Wiener index, Merrified–Simmons index, Kirchhoff index, and Hosoya index of polyphenyl chains were reported in [27–32] and the references therein.

**Figure 3:**
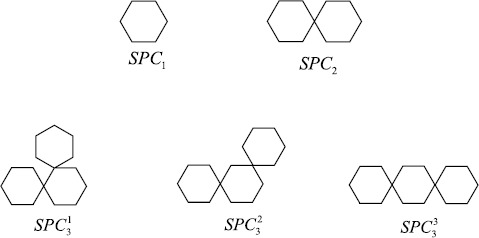
Spiro chains for $n=1,2,3$

**Figure 4:**
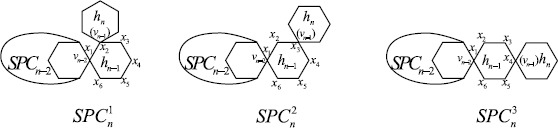
Three types of local arrangements in the spiro chain corresponding to a polyphenyl chain

A spiro chain of length *n*, denoted $\mathit{SPC}_{n}$, can be obtained from a polyphenyl chain $\mathit{PPC}_{n}$ by contracting each cut edge between each pair of consecutive hexagons in $\mathit{PPC}_{n}$. Figure 3 shows the unique spiro chains for $n=1,2$ and all spiro chains for $n=3$, and Figure 1(b) shows a general case, where $v_{n-1}$ is a vertex of $h_{n-1}$ in $\mathit{SPC}_{n-1}$. Similarly to the construction of a polyphenyl chain $\mathit{PPC}_{n}$, it is clear that $\mathit{SPC}_{n}$ is also not unique when $n\ge3$ and has three types of local arrangements, which are denoted by $\mathit{SPC}_{n}^{1}$, $\mathit{SPC}_{n}^{2}$, and $\mathit{SPC}_{n}^{3}$ (Figure 4). We may assume that getting an $\mathit{SPC}_{n}$ from a fixed $\mathit{SPC}_{n-1}$ is a random process. Namely, the probabilities of getting $\mathit{SPC}_{n}^{1}$, $\mathit{SPC}_{n}^{2}$, and $\mathit{SPC}_{n}^{3}$ from a fixed $\mathit{SPC}_{n-1}$ are $p_{1}$, $p_{2}$, and $1-p_{1}-p_{2}$, respectively. We also assume that the probabilities $p_{1}$ and $p_{2}$ are constants and independent of *n*, that is, the process described is a zeroth-order Markov process. After associating probabilities, such a spiro chain is called a *random spiro chain* and denoted by $\mathit{SPC}(n;p_{1},p_{2})$. For some contributions on spiro chains, the readers are referred to [27, 28, 33–35]. In 2015, Huang et al. [30] considered the expected value of the Kirchhoff index in a random spiro chain. For more results concerning other random chains, we refer to [36–42] and the references therein.

The rest of this paper is organized as follows. In Section 2, we present explicit formulae for the expected values of the *ABC* and *GA* indices of random spiro chains. Based on these formulae, we then give the average values of the *ABC* and *GA* indices with respect to the set of all spiro chains with *n* hexagons in Section 3 and make a comparison between the expected values of the *ABC* and *GA* indices in random spiro chains in Section 4.

## The *ABC* and *GA* indices in random spiro chains

In this section, we consider the *ABC* and *GA* indices in a random spiro chain. We keep the notation defined in Section 1. Let $\mathit{SPC}_{n}$ be the spiro chain obtained by attaching a new hexagon $h_{n}$ to $\mathit{SPC}_{n-1}$ as described in Figure 1(b). Assume that $h_{n}=x_{1}x_{2}x_{3}x_{4}x_{5}x_{6}$ as shown in Figure 2. Clearly, there are only $(2,2)$-, $(2,4)$-, and $(4,4)$-edges in a spiro chain $\mathit{SPC}_{n}$. By the definitions of the *ABC* and *GA* indices we can directly check that

3 $$ \mathit{ABC}(\mathit{SPC}_{n})=\frac{\sqrt{2}}{2}m_{22}(\mathit{SPC}_{n})+ \frac{\sqrt {2}}{2}m_{24}(\mathit{SPC}_{n})+\frac{\sqrt{6}}{4}m_{44}(\mathit{SPC}_{n}) $$

and

4 $$ \mathit{GA}(\mathit{SPC}_{n})=m_{22}(\mathit{SPC}_{n})+ \frac{2\sqrt {2}}{3}m_{24}(\mathit{SPC}_{n})+m_{44}(\mathit{SPC}_{n}). $$

Thus, to compute the *ABC* and *GA* indices of $\mathit{SPC}_{n}$, we just need to determine $m_{22}(\mathit{SPC}_{n})$, $m_{24}(\mathit{SPC}_{n})$, and $m_{44}(\mathit{SPC}_{n})$.

Recall that $\mathit{SPC}(n;p_{1},p_{2})$ is a random spiro chain of length *n*. Clearly, both $\mathit{ABC}(\mathit{SPC}(n; p_{1},p_{2}))$ and $\mathit{GA}(\mathit{SPC}(n;p_{1},p_{2}))$ are random variables. For convenience, denote their expected values by $E_{n}^{a}=E[\mathit{ABC}(\mathit{SPC}(n;p_{1},p_{2}))]$ and $E_{n}^{g}=E[\mathit{GA}(\mathit{SPC}(n;p_{1},p_{2}))]$, respectively.

We first give a formula for the expected value of the *ABC* index of a random spiro chain.

### Theorem 2.1

*Let* $\mathit{SPC}(n;p_{1},p_{2})$ *be a random spiro chain of length* $n\geq1$. *Then*

$$ E\bigl[\mathit{ABC}\bigl(\mathit{SPC}(n;p_{1},p_{2})\bigr)\bigr] = \biggl[ \biggl(\frac{\sqrt{6}}{4}-\frac{\sqrt{2}}{2} \biggr)p_{1}+3\sqrt {2} \biggr]n+ \biggl(\frac{\sqrt{2}}{2}-\frac{\sqrt{6}}{4} \biggr)p_{1}. $$

### Proof

When $n =1$, there is only one hexagon. So $E_{1}^{a}=6\times\frac{\sqrt{2}}{2}=3\sqrt{2}$.

When $n\geq2$, it is obvious that $m_{22}(\mathit{SPC}_{n})$, $m_{24}(\mathit{SPC}_{n})$, and $m_{44}(\mathit{SPC}_{n})$ depend on the three possible constructions as shown in Figure 3.

(i) If $\mathit{SPC}_{n-1}\rightarrow \mathit{SPC}_{n}^{1}$ with probability $p_{1}$, then we have

$$\begin{aligned} m_{22}\bigl(\mathit{SPC}_{n}^{1}\bigr)=m_{22}(\mathit{SPC}_{n-1})+3, m_{24}\bigl(\mathit{SPC}_{n}^{1}\bigr)=m_{24}(\mathit{SPC}_{n-1})+2 \end{aligned}$$

and

$$ m_{44}\bigl(\mathit{SPC}_{n}^{1}\bigr)=m_{44}(\mathit{SPC}_{n-1})+1. $$

Therefore by (3) we have

$$ \mathit{ABC}\bigl(\mathit{SPC}_{n}^{1}\bigr)=\mathit{ABC}(\mathit{SPC}_{n-1})+ \frac{5\sqrt{2}}{2}+ \frac{\sqrt{6}}{4}. $$

(ii) If $\mathit{SPC}_{n-1}\rightarrow \mathit{SPC}_{n}^{2}$ with probability $p_{2}$, then we have

$$\begin{aligned} m_{22}\bigl(\mathit{SPC}_{n}^{2}\bigr)=m_{22}(\mathit{SPC}_{n-1})+2, m_{24}\bigl(\mathit{SPC}_{n}^{2}\bigr)=m_{24}(\mathit{SPC}_{n-1})+4 \end{aligned}$$

and

$$ m_{44}\bigl(\mathit{SPC}_{n}^{2}\bigr)=m_{44}(\mathit{SPC}_{n-1}). $$

Therefore by (3) we have

$$ \mathit{ABC}\bigl(\mathit{SPC}_{n}^{2}\bigr)=\mathit{ABC}(\mathit{SPC}_{n-1})+3 \sqrt{2}. $$

(iii) If $\mathit{SPC}_{n-1}\rightarrow \mathit{SPC}_{n}^{3}$ with probability $1-p_{1}-p_{2}$, then we have

$$\begin{aligned} m_{22}\bigl(\mathit{SPC}_{n}^{3}\bigr)=m_{22}(\mathit{SPC}_{n-1})+2, m_{24}\bigl(\mathit{SPC}_{n}^{3}\bigr)=m_{24}(\mathit{SPC}_{n-1})+4 \end{aligned}$$

and

$$ m_{44}\bigl(\mathit{SPC}_{n}^{3}\bigr)=m_{44}(\mathit{SPC}_{n-1}). $$

Therefore by (3) we have

$$ \mathit{ABC}\bigl(\mathit{SPC}_{n}^{3}\bigr)=\mathit{ABC}(\mathit{SPC}_{n-1})+3 \sqrt{2}. $$

Thus we obtain

$$\begin{aligned} E_{n}^{a}&=E\bigl[\mathit{ABC}\bigl(\mathit{SPC}(n,p_{1},p_{2}) \bigr)\bigr] \\ &=p_{1}\mathit{ABC}\bigl(\mathit{SPC}_{n}^{1}\bigr)+p_{2}\mathit{ABC} \bigl(\mathit{SPC}_{n}^{2}\bigr)+(1-p_{1}-p_{2})\mathit{ABC} \bigl(\mathit{SPC}_{n}^{3}\bigr) \\ &=\mathit{ABC}(\mathit{SPC}_{n-1})+\biggl(\frac{\sqrt{6}}{4}-\frac{\sqrt{2}}{2} \biggr)p_{1}+3\sqrt{2}. \end{aligned}$$

Note that $E[E_{n}^{a}]=E_{n}^{a}$. Applying the expectation operator to the last equation, we get

5 $$ E_{n}^{a}=E_{n-1}^{a}+ \biggl(\frac{\sqrt{6}}{4}-\frac{\sqrt{2}}{2}\biggr)p_{1}+3\sqrt {2}\quad \mbox{for }n\ge2. $$

Since equation (5) is a first-order nonhomogeneous linear difference equation with constant coefficients. It is clear that the general solution of the homogeneous part of equation (5) is $E^{a}=c$, a constant.

Let $E^{a*}=an$ be a particular solution of equation (5). Substituting $E^{a*}$ into equation (5) and comparing the constant term, we have

$$ a=\biggl(\frac{\sqrt{6}}{4}-\frac{\sqrt{2}}{2}\biggr)p_{1}+3\sqrt{2}. $$

Consequently, the general solution of equation (5) is

$$ E_{n}^{a}=E^{a*}+E^{a}=E\bigl[\mathit{ABC} \bigl(\mathit{SPC}(n;p_{1},p_{2})\bigr)\bigr]= \biggl[\biggl( \frac{\sqrt{6}}{4}-\frac {\sqrt{2}}{2}\biggr)p_{1}+3\sqrt{2} \biggr]n+C \quad\mbox{for }n\ge1. $$

Substituting the initial condition, we obtain

$$ C= \biggl(\frac{\sqrt{2}}{2}-\frac{\sqrt{6}}{4} \biggr)p_{1}. $$

Therefore we have

$$ E_{n}^{a}= \biggl[ \biggl(\frac{\sqrt{6}}{4}- \frac{\sqrt{2}}{2} \biggr)p_{1}+3\sqrt {2} \biggr]n+ \biggl( \frac{\sqrt{2}}{2}-\frac{\sqrt{6}}{4} \biggr)p_{1}. $$

This completes the proof. □

We now give the formula for the expected value of the *GA* index of a random spiro chain.

### Theorem 2.2

*Let* $\mathit{SPC}(n;p_{1},p_{2})$ *be a random spiro chain of length* $n\geq1$. *Then*

$$ E\bigl[\mathit{GA}\bigl(\mathit{SPC}(n;p_{1},p_{2})\bigr)\bigr] = \biggl[ \biggl(2-\frac{4\sqrt{2}}{3} \biggr)p_{1}+2+\frac{8\sqrt{2}}{3} \biggr]n+ \biggl(\frac{4\sqrt{2}}{3}-2 \biggr)p_{1}+ \biggl(4-\frac{8\sqrt {2}}{3} \biggr). $$

### Proof

When $n=1$, there is only one hexagon. So $E_{1}^{g}=E[\mathit{GA}(\mathit{SPC}(1;p_{1},p_{2}))]=6$.

When $n\geq2$, it is obvious that $m_{22}(\mathit{SPC}_{n})$, $m_{24}(\mathit{SPC}_{n})$, and $m_{44}(\mathit{SPC}_{n})$ depend on the three possible constructions as shown in Figure 3.

(i) If $\mathit{SPC}_{n-1}\rightarrow \mathit{SPC}_{n}^{1}$ with probability $p_{1}$, then we get

$$\begin{aligned} m_{22}\bigl(\mathit{SPC}_{n}^{1}\bigr)=m_{22}(\mathit{SPC}_{n-1})+3, m_{24}\bigl(\mathit{SPC}_{n}^{1}\bigr)=m_{24}(\mathit{SPC}_{n-1})+2 \end{aligned}$$

and

$$ m_{44}\bigl(\mathit{SPC}_{n}^{1}\bigr)=m_{44}(\mathit{SPC}_{n-1})+1. $$

Therefore by (4) we have

$$ \mathit{GA}\bigl(\mathit{SPC}_{n}^{1}\bigr)=\mathit{GA}(\mathit{SPC}_{n-1})+4+ \frac{4\sqrt{2}}{3}. $$

(ii) If $\mathit{SPC}_{n-1}\rightarrow \mathit{SPC}_{n}^{2}$ with probability $p_{2}$, then we get

$$\begin{aligned} m_{22}\bigl(\mathit{SPC}_{n}^{2}\bigr)=m_{22}(\mathit{SPC}_{n-1})+2, m_{24}\bigl(\mathit{SPC}_{n}^{2}\bigr)=m_{24}(\mathit{SPC}_{n-1})+4 \end{aligned}$$

and

$$ m_{44}\bigl(\mathit{SPC}_{n}^{2}\bigr)=m_{44}(\mathit{SPC}_{n-1}). $$

Therefore by (4) we have

$$ \mathit{GA}\bigl(\mathit{SPC}_{n}^{2}\bigr)=\mathit{GA}(\mathit{SPC}_{n-1})+2+ \frac{8\sqrt{2}}{3}. $$

(iii) If $\mathit{SPC}_{n-1}\rightarrow \mathit{SPC}_{n}^{3}$ with probability $1-p_{1}-p_{2}$, then we have

$$\begin{aligned} m_{22}\bigl(\mathit{SPC}_{n}^{3}\bigr)=m_{22}(\mathit{SPC}_{n-1})+2, m_{24}\bigl(\mathit{SPC}_{n}^{3}\bigr)=m_{24}(\mathit{SPC}_{n-1})+4 \end{aligned}$$

and

$$ m_{44}\bigl(\mathit{SPC}_{n}^{3}\bigr)=m_{44}(\mathit{SPC}_{n-1}). $$

Therefore by (4) we have

$$ \mathit{GA}\bigl(\mathit{SPC}_{n}^{3}\bigr)=\mathit{GA}(\mathit{SPC}_{n-1})+2+ \frac{8\sqrt{2}}{3}. $$

Thus we obtain

$$\begin{aligned} E_{n}^{g}&=E\bigl[\mathit{GA}\bigl(\mathit{SPC}(n,p_{1},p_{2}) \bigr)\bigr] \\ &=p_{1}\mathit{GA}\bigl(\mathit{SPC}_{n}^{1}\bigr)+p_{2}\mathit{GA} \bigl(\mathit{SPC}_{n}^{2}\bigr)+(1-p_{1}-p_{2})\mathit{GA} \bigl(\mathit{SPC}_{n}^{3}\bigr) \\ &=\mathit{GA}(\mathit{SPC}_{n-1})+\biggl(2-\frac{4\sqrt{2}}{3}\biggr)p_{1}+ \biggl(2+\frac{8\sqrt{2}}{3}\biggr). \end{aligned}$$

Note that $E[E_{n}^{g}]=E_{n}^{g}$. Applying the expectation operator to the last equation, we get

6 $$ E_{n}^{g}=E_{n-1}^{g}+ \biggl(2-\frac{4\sqrt{2}}{3}\biggr)p_{1}+\biggl(2+\frac{8\sqrt{2}}{3} \biggr),\quad \mbox{for }n\ge2. $$

Since equation (6) is a first-order nonhomogeneous linear difference equation with constant coefficients, it is clear that the general solution of the homogeneous part of equation (6) is $E^{g}=c$, a constant.

Let $E^{g*}=an$ be a particular solution of equation (6). Substituting $E^{g*}$ into equation (6) and comparing the constant term, we have

$$ a=\biggl(2-\frac{4\sqrt{2}}{3}\biggr)p_{1}+\biggl(2+\frac{8\sqrt{2}}{3} \biggr). $$

Consequently, the general solution of equation (6) is

$$ \begin{aligned} E_{n}^{g}&=E^{g*}+E^{g}=E\bigl[\mathit{GA} \bigl(\mathit{SPC}(n;p_{1},p_{2})\bigr)\bigr]\\ &= \biggl[\biggl(2- \frac{4\sqrt {2}}{3}\biggr)p_{1}+\biggl(2+\frac{8\sqrt{2}}{3}\biggr) \biggr]n+C \quad\mbox{for }n\ge1. \end{aligned} $$

Substituting the initial condition, we obtain

$$ C= \biggl(\frac{4\sqrt{2}}{3}-2 \biggr)p_{1}+ \biggl(4- \frac{8\sqrt {2}}{3} \biggr). $$

Therefore we have

$$ E_{n}^{g}= \biggl[\biggl(2-\frac{4\sqrt{2}}{3} \biggr)p_{1}+\biggl(2+\frac{8\sqrt{2}}{3}\biggr) \biggr]n+ \biggl( \frac{4\sqrt{2}}{3}-2 \biggr)p_{1}+ \biggl(4-\frac{8\sqrt {2}}{3} \biggr), $$

and the proof is completed. □

In Theorems 2.1 and 2.2, we observe that both $E[\mathit{ABC}(\mathit{SPC}(n;p_{1},p_{2}))]$ and $E[\mathit{GA}(\mathit{SPC}(n; p_{1},p_{2}))]$ are asymptotic to *n* and linear in $p_{1}$. Therefore, by Theorems 2.1 and 2.2 we can easily obtain the *ABC* and *GA* indices of spiro meta-chain $O_{n}$, spiro orth-chain $M_{n}$, and spiro para-chain $P_{n}$ (defined in [30]).

### Corollary 2.3

*The* *ABC* *indices of the spiro meta*-*chain* $O_{n}$, *the spiro orth*-*chain* $M_{n}$, *and the spiro para*-*chain* $P_{n}$ *are*

$$ \mathit{ABC}(O_{n})= \biggl(\frac{\sqrt{6}}{4}+\frac{5\sqrt{2}}{2} \biggr)n+ \frac {\sqrt{2}}{2}-\frac{\sqrt{6}}{4} $$

*and*

$$ \mathit{ABC}(M_{n})=\mathit{ABC}(P_{n})=3\sqrt{2}n. $$

### Corollary 2.4

*The* *GA* *indices of the spiro meta*-*chain* $O_{n}$, *the spiro orth*-*chain* $M_{n}$, *and the spiro para*-*chain* $P_{n}$ *are*

$$ \mathit{GA}(O_{n})= \biggl(4+\frac{4\sqrt{2}}{3} \biggr)n+2-\frac{4\sqrt{2}}{3} $$

*and*

$$ \mathit{GA}(M_{n})=\mathit{GA}(P_{n})= \biggl(2+\frac{8\sqrt{2}}{3} \biggr)n+4-\frac{8\sqrt{2}}{3}. $$

## The average values of *ABC* and *GA* indices

In this section, we present the average values of the *ABC* and *GA* indices with respect to the set of all spiro chains with *n* hexagons.

Let $\mathscr{SP}_{n}$ be the set of all spiro chains with *n* hexagons. The average values of the *ABC* and *GA* indices of $\mathscr{SP}_{n}$ are defined by

$$\mathit{ABC}_{\mathrm{avr}}(\mathscr{SP}_{n})=\frac{1}{ \vert \mathscr{SP}_{n} \vert }\sum _{G\in\mathscr{SP}_{n}}\mathit{ABC}(G) $$

and

$$\mathit{GA}_{\mathrm{avr}}(\mathscr{SP}_{n})=\frac{1}{ \vert \mathscr{SP}_{n} \vert }\sum _{G\in\mathscr{SP}_{n}}\mathit{GA}(G), $$

respectively. In fact, this is the population mean of the *ABC* and *GA* indices of all elements in $\mathscr{SP}_{n}$. Since every element occurring in $\mathscr{SP}_{n}$ has the same probability, we have $p_{1}=p_{2}=1-p_{1}-p_{2}$. Thus we can apply Theorems 2.1 and 2.2 by putting $p_{1}=p_{2}=1-p_{1}-p_{2}=\frac{1}{3}$ and obtain the following result.

### Theorem 3.1

*The average values of the* *ABC* *and* *GA* *indices with respect to* $\mathscr{SP}_{n}$ *are*

$$ \mathit{ABC}_{\mathrm{avr}}(\mathscr{SP}_{n})= \biggl(\frac{\sqrt {6}}{12}+ \frac{17\sqrt{2}}{6} \biggr)n +\frac{\sqrt{2}}{6}-\frac{\sqrt{6}}{12} $$

*and*

$$ \mathit{GA}_{\mathrm{avr}}(\mathscr{SP}_{n})= \biggl(\frac{8}{3}+ \frac {20\sqrt{2}}{9} \biggr)n +\frac{10}{3}-\frac{20\sqrt{2}}{9}. $$

From Theorem 3.1, as well as from Corollaries 2.3 and 2.4, it is no difficult to see that the average values of the *ABC* and *GA* indices with respect to $\{O_{n}, M_{n},P_{n}\}$ are

$$ \frac{\mathit{ABC}(O_{n})+\mathit{ABC}(M_{n})+\mathit{ABC}(P_{n})}{3}= \biggl(\frac{\sqrt {6}}{12}+\frac{17\sqrt{2}}{6} \biggr)n + \frac{\sqrt{2}}{6}-\frac{\sqrt{6}}{12} $$

and

$$ \frac{\mathit{GA}(O_{n})+\mathit{GA}(M_{n})+\mathit{GA}(P_{n})}{3}= \biggl(\frac {8}{3}+\frac{20\sqrt{2}}{9} \biggr)n + \frac{10}{3}-\frac{20\sqrt{2}}{9}, $$

which indicate that the average values of the *ABC* and *GA* indices with respect to $\mathscr{SP}_{n}$ are exactly equal to the average values of the *ABC* and *GA* indices with respect to $\{ O_{n}, M_{n},P_{n}\}$, respectively.

## A comparison between the expected values of *ABC* and *GA* indices

Das and Trinajstić [15] compared the first *GA* index and *ABC* index for chemical trees, molecular graphs, and simple graphs with some restricted conditions. Recently, Ke [40] also compared the expected values of the *GA* index and *ABC* index for a random polyphenyl chain. Using Theorems 2.1 and 2.2, we now make a comparison between the expected values for the *ABC* and *GA* indices of a random spiro chain with the same probability $p_{i}$ ($i=1,2$).

### Theorem 4.1

*Let* $\mathit{SPC}(n;p_{1},p_{2})$ *be a random spiro chain with* *n* *hexagons*. *Then*

$$ E \bigl[\mathit{GA}(\mathit{SPC}(n;p_{1},p_{2}) \bigr]>E \bigl[\mathit{ABC}(\mathit{SPC}(n;p_{1},p_{2}) \bigr]. $$

### Proof

When $n=1$, it is clear that

$$ E \bigl[\mathit{GA}(\mathit{SPC}(1;p_{1},p_{2}) \bigr]=6>3\sqrt{2}=E \bigl[\mathit{ABC}(\mathit{SPC}(1;p_{1},p_{2}) \bigr]. $$

When $n\geq2$, by Theorems 2.1 and 2.2 we have

$$\begin{aligned} &E \bigl[\mathit{GA}(\mathit{SPC}(n;p_{1},p_{2}) \bigr]-E \bigl[\mathit{ABC}(\mathit{SPC}(n;p_{1},p_{2}) \bigr] \\ &\quad= \biggl[\biggl(2-\frac{\sqrt{6}}{4}-\frac{4\sqrt{2}}{3}+\frac{\sqrt {2}}{2} \biggr)p_{1}+2+\frac{8\sqrt{2}}{3}-3\sqrt{2} \biggr]n\\ &\qquad {}+ \biggl( \frac{4\sqrt {2}}{3}-2-\frac{\sqrt{2}}{2}+\frac{\sqrt{6}}{4} \biggr)p_{1}+4- \frac{8\sqrt{2}}{3}. \end{aligned}$$

Noting that $2-\frac{\sqrt{6}}{4}-\frac{4\sqrt{2}}{3}+\frac{\sqrt{2}}{2}>0$ and $0\leq p_{1}\leq1$, we get

$$\begin{aligned} &E \bigl[\mathit{GA}(\mathit{SPC}(n;p_{1},p_{2}) \bigr]-E \bigl[\mathit{ABC}(\mathit{SPC}(n;p_{1},p_{2}) \bigr] \\ &\quad\geq \biggl(2+\frac{8\sqrt{2}}{3}-3\sqrt{2} \biggr)n+ \biggl( \frac{4\sqrt {2}}{3}-2-\frac{\sqrt{2}}{2}+\frac{\sqrt{6}}{4} \biggr)\times1+4- \frac{8\sqrt{2}}{3} \\ &\quad= \biggl(2-\frac{\sqrt{2}}{3} \biggr)n+2+\frac{\sqrt{6}}{4}- \frac {11\sqrt{2}}{6} \\ &\quad\geq \biggl(2-\frac{\sqrt{2}}{3} \biggr)\times2+2+\frac{\sqrt {6}}{4}- \frac{11\sqrt{2}}{6} \\ &\quad>0, \end{aligned} $$

as desired. This completes the proof. □

Theorem 4.1 states that the expected value of the *ABC* index is less than the expected value of the *GA* index for a random spiro chain, which is similar to the result for a random polyphenyl chain [40].

## Conclusions

In this paper, we mainly study the *ABC* and *GA* indices in random spiro chains. Firstly, we study explicit formulae for the expected values of the *ABC* and *GA* indices in random spiro chains, similar to the results obtained in [30, 33]. Secondly, we present the average values of the *ABC* and *GA* indices with respect to the set of all spiro chains with *n* hexagons. Finally, we compare the expected values of the *ABC* and *GA* indices in random spiro chains and show that the expected value of the *ABC* index is less than the expected value of the *GA* index.
